# Tension Pneumothorax Following Pneumocystis jirovecii Pneumonia

**DOI:** 10.7759/cureus.6799

**Published:** 2020-01-28

**Authors:** Kulachanya Suwanwongse, Nehad Shabarek

**Affiliations:** 1 Internal Medicine, Lincoln Medical Center, New York City, USA

**Keywords:** pjp, pneumothorax, pneumocystis, pneumonia, hiv, aids, pcp, case report

## Abstract

Pneumocystis jirovecii pneumonia (PJP) is one of the most common causes of respiratory failure in patients with human immunodeficiency virus (HIV) infection, for which the mortality rate is approximately 10%. Spontaneous pneumothorax as a presentation of PJP has been reported with rising frequency, but tension pneumothorax as a presentation of PJP is rare. We reported a case of a middle-aged male with HIV infection who presented to our hospital with acute worsening shortness of breath and was later diagnosed with tension pneumothorax due to PJP. Physicians should suspect pneumothorax in patients with a clinical presentation of PJP who show rapid progressive respiratory deterioration.

## Introduction

Pneumocystis jirovecii pneumonia (PJP) is a devastating opportunistic infection in patients with acquired immune deficiency syndrome (AIDS), which can lead to respiratory failure and death if untreated. Spontaneous pneumothorax is a catastrophic complication of PJP, requiring urgent management to limit morbidity and mortality [1]. We presented the unusual case of tension pneumothorax arising from PJP, in which we found only two previous cases were reported in PubMed databases. Clinicians should be aware that patients with PJP may present with a clinical presentation of pneumothorax, and patients with HIV infection who develop pneumothorax should be considered for possible PJP.

## Case presentation

**Figure 1:**
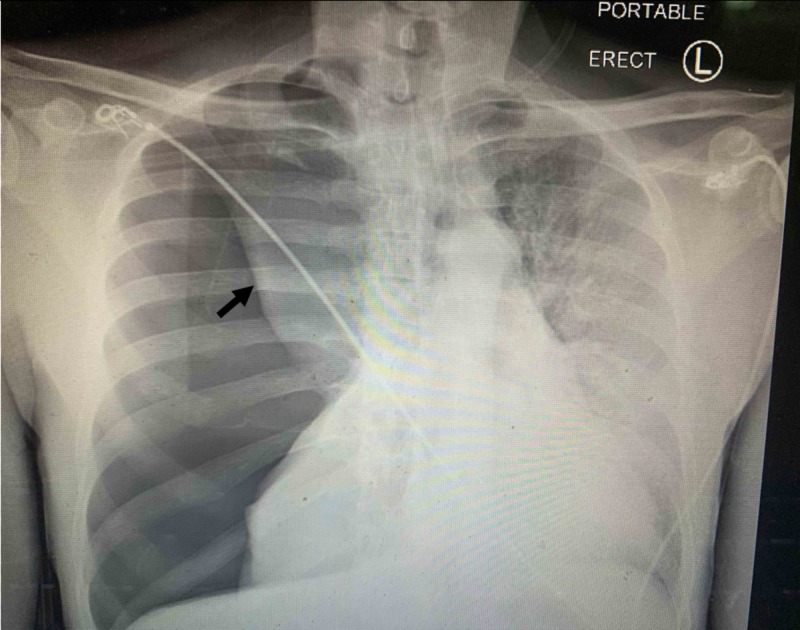
Chest X-ray showed massive right-sided tension pneumothorax with collapse of the right lung (arrow) and leftward shift of the mediastinum

A 46-year-old male with a past medical history of HIV infection, non-compliance with highly active antiretroviral therapy (HAART), presented to our emergency department with a complaint of severe worsening shortness of breath. He traveled to North Carolina a week before when he started having a gradual onset of low-grade fever, dry cough, and sore throat. He began to have shortness of breath three days before admission but abrupt worsening a few hours before. On initial evaluation, he had high blood pressure, systolic blood pressure of 170 mmHg and diastolic blood pressure of 120 mmHg, tachycardia with heart rate 120 beats per minute, tachypnea, and hypoxemia with oxygen saturation between 80% and 90% on a 15-liter non-rebreather oxygen mask. On examination, the patient looked uncomfortable and agitated. He sat upright, spoke in short sentences, and used respiratory accessory muscles. His breath sound was absent at the right chest. Chest X-ray showed massive right-sided tension pneumothorax with the collapse of the right lung and leftward shift of the mediastinum, as demonstrated in Figure 1.

**Figure 2:**
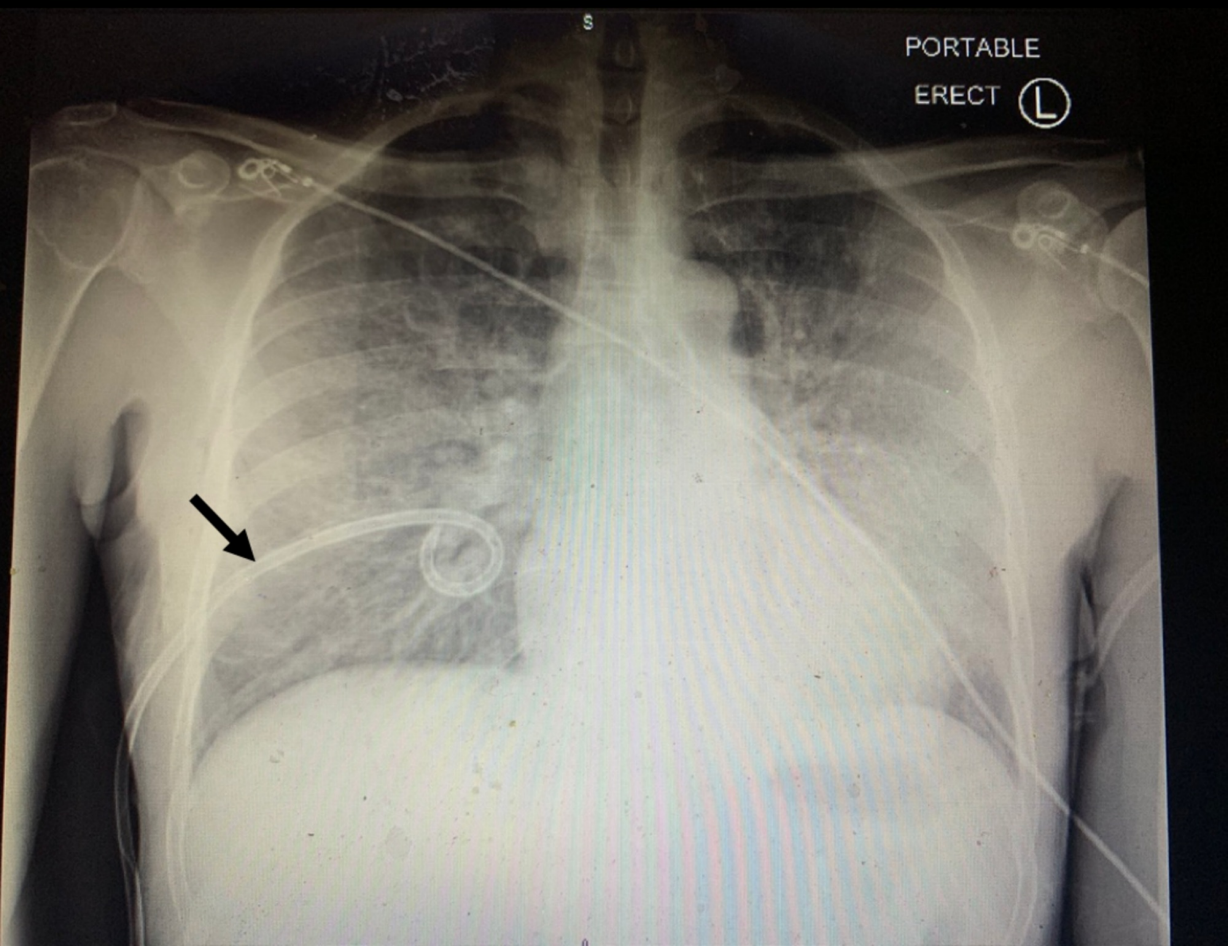
Chest X-ray following the chest tube placement (arrow) showed resolution of the pneumothorax

The surgery team placed a chest tube; however, the patient's symptoms did not improve, and the decision was made to intubate him and transfer to the medical intensive care unit. Chest X-ray following the chest tube placement was demonstrated in Figure 2, which showed resolution of the pneumothorax.

Blood tests were remarkable for markedly elevated lactate dehydrogenase at 866U/L. His CD4 count was 17. Bronchoalveolar lavage confirmed a positive Gomori methenamine silver stain for PJP. He received treatment with sulfamethoxazole/trimethoprim and prednisolone. His hospital course was complicated with Clostridium difficile colitis. He was discharged home on a tracheostomy tube due to prolong intubation after 57 days of admission with successful removal of the tracheostomy tube a month later. 

## Discussion

PJP occurs predominantly in patients with HIV infection with a CD4 count of less than 200. Although the incidence of PJP has been decreasing since the advent of HAART, PJP remains the most common opportunistic infection in HIV patients and accounts for the highest proportion of AIDS-related deaths [2]. Patients with PJP always present with subacute to chronic low-grade fever, cough, and progressive exertional shortness of breath [3]. Sudden onset of new or worsening respiratory symptoms in patients with PJP may indicate the development of PJP-related complications, including pneumothorax. 

HIV patients with active or previous Pneumocystis infection were found to have a higher risk of developing a pneumothorax [4]. Spontaneous pneumothorax following PJP can be explained by three possible mechanisms. First, PJP leads to severe inflammation of lung tissue, resulting in necrotizing alveolitis and a replacement of the normal lung parenchymal tissue with cysts and pneumatoceles, which later rupture and release air into the pleural space leading to pneumothorax [1]. Second, PJP may cause local subpleural necrosis of lung tissue leading to cavitation and formation of bronchopleural fistula and subsequently a pneumothorax [5]. Third, severe interstitial inflammation and later fibrosis from PJP can lead to a lung contracture with seepage of air from visceral pleura to the pleural space [6]. Besides, pneumothorax in patients with PJP may arise from diagnostic and therapeutic complications, including iatrogenic pneumothorax from bronchoscopy, barotrauma from mechanical ventilation, and apical disease changes in aerosolized pentamidine [7]. 

Our case presented with subacute low-grade fever and respiratory symptoms, and later reported abrupt worsening of shortness of breath due to tension pneumothorax. The diagnosis of tension pneumothorax is based on clinical presentation and physical examination. To prevent morbidity and mortality of this condition, decompressive treatment for tension pneumothorax should not be delayed for the chest x-ray confirmation which is a pitfall in our case. It is recognized that PJP can present with pneumothorax but clinicians should be aware that PJP can also lead to rare but life-threatening tension pneumothorax. 

## Conclusions

Pneumothorax should be included in a differential diagnosis in patients with suspected PJP, who developed sudden onset of respiratory deterioration. Tension pneumothorax is a clinical diagnosis, and treatment should not be delayed for radiologic confirmation. 
